# Supplementary material for: Antibiotic Resistance of Selected Bacteria after Treatment of the Supragingival Biofilm with Subinhibitory Chlorhexidine Concentrations
Source: Antibiotics (Basel). 2022 Oct 17;11(10):1420. doi: 10.3390/antibiotics11101420 (PMC9598507; doi:10.3390/antibiotics11101420)
Supplement: Supplementary file 1 [file antibiotics-11-01420-s001.zip › antibiotics-1939022-supplementary.pdf]

Supporting Information

Antibiotic Resistance of Selected Bacteria after Treatment of the Supragingival Biofilm with Subinhibitory Chlorhexidine Concentrations

Robin Früh [1,*], Annette Anderson [1], Fabian Cieplik [2], Elmar Hellwig [1], Annette Wittmer [3], Kirstin Vach [4] and Ali Al-Ahmad [1]

[1] Department of Operative Dentistry & Periodontology, Faculty of Medicine, University of Freiburg, 79106 Freiburg, Germany
[2] Department of Conservative Dentistry and Periodontology, University Hospital Regensburg, 93053 Regensburg, Germany
[3] Department of Microbiology and Hygiene, Institute of Medical Microbiology and Hygiene, Medical Center, University of Freiburg, 79085 Freiburg, Germany
[4] Institute of Medical Biometry and Statistics, Faculty of Medicine, 79104 Freiburg, Germany
* Correspondence: robin.frueh@uniklinik-freiburg.de

Table S1. Exact rating-values of the MALDI-TOF-analysis. Values > 2 show a reliable genus and species identification, Values = 2.0-1.7 show a probable genus and species identification, Values < 1.7 shows an insufficient identification.

Title	Genus	Species	MALDI-Rating
**P1 - Org**	*Streptococcus*	*oralis*	2.16
**P1 - Passaged without CHX**	*Streptococcus*	*oralis*	2.15
**P1 - Passaged with CHX**	*Streptococcus*	*oralis*	2.09
**P2 - Org**	*Streptococcus*	*oralis*	2.21
**P2 - Passaged without CHX**	*Streptococcus*	*oralis*	2.15
**P2 - Passaged with CHX**	*Streptococcus*	*oralis*	2.13
**P3 - Org**	*Streptococcus*	*oralis*	2.33
**P3 - Passaged without CHX**	*Streptococcus*	*oralis*	2.02
**P3 - Passaged with CHX**	*Streptococcus*	*oralis*	2.14
**P4 - Org**	*Streptococcus*	*oralis*	2.32
**P4 - Passaged without CHX**	*Streptococcus*	*oralis*	2.01
**P4 - Passaged with CHX**	*Streptococcus*	*oralis*	2.15
**P5 - Org**	*Streptococcus*	*oralis*	2.19
**P5 - Passaged without CHX**	*Streptococcus*	*oralis*	2.2
**P5 - Passaged with CHX**	*Streptococcus*	*oralis*	2.09
**P6 - Org**	*Streptococcus*	*oralis*	2.16
**P6 - Passaged without CHX**	*Streptococcus*	*oralis*	2.14
**P6 - Passaged with CHX**	*Streptococcus*	*oralis*	2.13
**P5 - Org**	*Granulicatella*	*adiacens*	2.23
**P5 - Passaged with CHX**	*Granulicatella*	*adiacens*	2.2

Table S2. The actual values of Antibiotic susceptibility of the isolates (in µg/mL). (a) P1- *S. oralis* (b) P2- *S. oralis* (c) P3- *S. oralis* (d) P4- *S. oralis* (e) P5- *S. oralis* (f) P6- *S. oralis* (g) P5- *G. adiacens* .

**(a) Proband 1 - *S. oralis***			
**Antibiotic**	**Org-MIC(µg/mL)**	**CHX-MIC(µg/mL)**	**Without CHX-MIC(µg/mL)**
Erythromycin	3	6	2
Clindamycin	0.064	0.047	0.094
Penicillin G	0.094	0.094	0.094
Ampicillin	0.032	0.19	0.125
Amoxicillin	0.016	0.094	0.064
Gentamicin	32	16	16
Tetracycline	32	0.25	0.25
Vancomycin	0.25	0.38	0,5
Meropenem	0.047	0.094	0.064
Ciprofloxacin	2	2	3
Cefuroxime	0.25	1	1.5
Tigecycline	0.094	0.032	0.064
Moxifloxacin	0.19	0.125	0.25
**(b) Proband 2 - *S. oralis***			
**Antibiotic**	**Org-MIC(µg/mL)**	**CHX-MIC(µg/mL)**	**Without CHX-MIC(µg/mL)**
Erythromycin	0.094	6	0.064
Clindamycin	0.064	0.094	0.064
Penicillin G	0.064	0.064	0.032
Ampicillin	0.023	0.016	0.047
Amoxicillin	0.016	0.023	0.032
Gentamicin	16	16	16
Tetracycline	0.19	0.25	0.125
Vancomycin	0.38	0.25	0.5
Meropenem	0.032	0.047	0.064
Ciprofloxacin	4	12	4
Cefuroxime	0.125	0.094	0.19
Tigecycline	0.064	0.064	0.064
Moxifloxacin	0.25	0.25	0.125
**(c) Proband 3 - *S. oralis***			
**Antibiotic**	**Org-MIC(µg/mL)**	**CHX-MIC(µg/mL)**	**Without CHX-MIC(µg/mL)**
Erythromycin	256	3	2
Clindamycin	256	0.064	0.047
Penicillin G	0.023	0.032	0.032
Ampicillin	0.032	0.032	0.032
Amoxicillin	0.032	0.032	0.023
Gentamicin	6	16	16
Tetracycline	16	0.25	0.19
Vancomycin	0.25	0.5	0.5
Meropenem	0.032	0.047	0.047
Ciprofloxacin	0.75	4	3
Cefuroxime	0.19	0.125	0.125
Tigecycline	0.016	0.047	0.047
Moxifloxacin	0.064	0.125	0.19
**(d) Proband 4 - *S. oralis***			
**Antibiotic**	**Org-MIC(µg/mL)**	**CHX-MIC(µg/mL)**	**Without CHX-MIC(µg/mL)**
Erythromycin	2	4	6
Clindamycin	0.064	0.094	0.125
Penicillin G	0.023	0.032	0.016
Ampicillin	0.023	0.047	0.016
Amoxicillin	0.023	0.032	0.032

Gentamicin	16	16	16
Tetracycline	0.19	0.25	0.19
Vancomycin	0.38	0.5	0.38
Meropenem	0.032	0.064	0.023
Ciprofloxacin	1.5	4	3
Cefuroxime	0.094	0.125	0.047
Tigecycline	0.047	0.047	0.064
Moxifloxacin	0.19	0.19	0.25

(e) Proband 5 - *S. oralis*

**Antibiotic**	**Org-MIC(µg/mL)**	**CHX-MIC(µg/mL)**	**Without CHX-MIC(µg/mL)**
Erythromycin	0.094	3	4
Clindamycin	0.064	0.064	0.064
Penicillin G	0.016	0.047	0.25
Ampicillin	0.047	0.032	0.5
Amoxicillin	0.032	0.023	0.38
Gentamicin	16	16	4
Tetracycline	0.19	64	24
Vancomycin	0.5	0.38	0.25
Meropenem	0.016	0.032	0.032
Ciprofloxacin	2	4	1.5
Cefuroxime	0.125	0.125	0.5
Tigecycline	0.064	0.094	0.047
Moxifloxacin	0.094	0.19	0.25

(f) Proband 6 - *S. oralis*

**Antibiotic**	**Org-MIC(µg/mL)**	**CHX-MIC(µg/mL)**	**Without CHX-MIC(µg/mL)**
Erythromycin	0.094	0.125	2
Clindamycin	0.064	0.094	0.094
Penicillin G	0.023	0.023	0.032
Ampicillin	0.047	0.032	0.047
Amoxicillin	0.047	0.023	0.032
Gentamicin	24	16	32
Tetracycline	0.19	0.25	0.19
Vancomycin	0.5	0.5	0.5
Meropenem	0.064	0.032	0.047
Ciprofloxacin	4	1.5	3
Cefuroxime	0.125	0.094	0.19
Tigecycline	0.094	0.094	0.047
Moxifloxacin	0.38	0.094	0.19

(g) Proband 5 – *G. adiacens*

**Antibiotic**	**Org-MIC(µg/mL)**	**CHX-MIC(µg/mL)**
Erythromycin	0.19	>256
Clindamycin	0.19	>256
Penicillin G	0.008	0.094
Ampicillin	<0.016	0.047
Amoxicillin	<0.016	0.064
Gentamicin	2	3
Tetracycline	0.5	16
Vancomycin	0.38	0.75
Meropenem	0.047	0.094
Ciprofloxacin	0.19	2
Cefuroxime	0.016	4
Tigecycline	0.064	0.094
Moxifloxacin	0.064	0.25
